# Home-Drying Operation Effect on Moisture Content, Electric Energy Consumption, Ascorbic Acid, Total Polyphenol Content, and Color of Sliced “Fuji” Apples

**DOI:** 10.1155/2023/9996331

**Published:** 2023-11-23

**Authors:** Jeanethe Monsalves, Erick Scheuermann

**Affiliations:** ^1^Undergraduate Program Industrial Civil Engineering mention in Bioprocesses, Faculty of Engineering and Sciences, Universidad de La Frontera, Temuco, Chile; ^2^Chemical Engineering Department, Center of Food Biotechnology and Bioseparations (BIOREN-UFRO) and CIBAMA, Universidad de La Frontera, Temuco, Chile

## Abstract

A home dehydrator allows obtaining dried apples that are beneficial to human health, but its operations will affect the chemical and organoleptic quality of this fruit. In this study, the effect of the drying temperature and mass load of sliced fresh “Fuji” apples in a home dehydrator was evaluated with regard to moisture content, electric energy consumption, ascorbic acid, total polyphenol content, and color of the dried fruit. Fresh “Fuji” apples were cut to obtain a uniform slice with a thickness of 4 mm and diameter from 60 to 75 mm. A home dehydrator was operated at 50 and 70°C (nominal temperatures), with a total sliced apple load of 250 and 500 g, uniformly distributed in five trays. Drying was carried out for 7 hours, and every hour, the trays were rotated, changing their position from the top to the bottom. Only the middle tray was always kept in the same position. As result, the level of nominal temperature (50/70°C) was not reached for any of the trays, regardless of the mass load (250/500 g) in the home dehydrator. The temperature average for fruits dried in trays of the home dehydrator that were rotated (top and bottom) and kept in the same position (middle) did not differ (*p* > 0.05) among them. At the end of drying, the apple treatment at 50°C/250 g, 50°C/500 g, 70°C/250 g, and 70°C/500 g reached 23.1, 26.2, 4.3, and 4.5% (w.b.), respectively. The drying conditions at nominal 50°C favored the quality of the dried sliced apples with regard to ascorbic acid and total polyphenol content; however, the treatment at nominal 70°C produced less variation in color with respect to the fresh fruit. The home dehydrator allowed obtaining sliced dried “Fuji” apples that adequately preserve the ascorbic acid, total polyphenol, and color with respect to the fresh fruit.

## 1. Introduction

Convective hot air drying is the most common method for fruit dehydration [1], and more than 85% of industrial dryers are convective with hot air [2]. On the other hand, several companies have been manufacturing home dehydrators, and interest in the use of this food preservation technology on a smaller scale has grown in the last years [3–6]. These sanitary dehydrators could be intended for home use or small businesses for drying several foods [7–12].

The worldwide consumption of dried apples as a snack (slices, cubes, etc.) is popular due to its photochemical components that are beneficial to human health and attractive organoleptic quality [13, 14]. Apple drying using home equipment allows the fruit preservation and avoids waste. Several quality parameters undergo changes during drying and storage and suffered changes in TPC, TFC, ORAC, and TEAC values [15, 16].

The operating conditions of home dehydrators vary according to the cylindrical or cubic shape of the equipment, number of trays, temperature range, and air circulation. These drying conditions affect the chemical, microbiological, and organoleptic characteristics of the dried food [7–12]. Quality parameters undergo several changes during drying and storage. Major quality parameters associated with dried food products include color, visual appeal, shape of the product, flavour, microbial load, retention of nutrients, porosity-bulk density, and texture [1, 17–20]. During apple chip production, ascorbic acid suffered thermal degradation and lower TPC, TFC, ORAC, and TEAC values, and hydroxymethylfurfural content increased with drying time and temperature [21]. Drying of two apple cultivars affected acidity, sugar content, and color, and the products turned less hard and cohesive [19]. Finally, the operating conditions at the home-drying level influence the energy expenditure that is relevant for the cost of the household apple drying [12, 20, 22]. The adequate operative condition of the home dehydrator would allow obtaining good quality dried apples with reasonable energy expenditure. Hence, the objective of this work was to evaluate the effect of the drying temperature and mass load of sliced fresh “Fuji” apples in a home dehydrator on moisture content, electric energy consumption, ascorbic acid, total polyphenol content, and color of the dried fruit.

## 2. Materials and Methods

### 2.1. Plant Material and Sample Preparation

“Fuji” variety apples were purchased at a local fruit store (Jireh Frutería y Verdulería) of Temuco city (Chile), transported immediately to the Food Science Laboratory at the Universidad de La Frontera, and used in each experiment. All fresh apples used were suitable for consumption, without any damage or deterioration on their surface. The fruit was stored in a temperature-controlled chamber (Archiclima, Chile) at 5°C for a maximum of one week before it was used for experimentation.

#### 2.1.1. Slicing

Fresh “Fuji” apples were washed with drinking water, the surface water was removed with an absorbent paper towel, and then, they were cut using a slicer (Model Mandolina, Attimo, Chile) to obtain a uniform circular slice with a thickness of 4 mm and diameter from 60 to 75 mm. The mass percentage with which the home dehydrator was loaded was characterized according to the circular sliced apples. For both 250 g and 500 g used in this research (points 2.1.2. and 2.3.6.), 23.6% of this mass were slices with a diameter of 60 to 65 mm, 23.4% from 66 to 70 mm, and 53% from 71 to 75 mm.

Regarding the average diameters and standard deviation (data provided in parentheses), the range from 60 to 65 mm was 6.2 mm (±0.2 mm), from 66 to 70 mm was 6.9 mm (±0.1 mm), and from 71 to 75 mm was 7.3 mm (±0.1 mm).

For cut apple slices that had a diameter in the range of 60 to 65 mm, the average mass per slice was 6.8 g (±0.9 g); for 66 to 70 mm, it was 8.6 g (±1.1 g); and for 71 to 75 mm, it was 10.2 g (±1.0 g).

Finally, the average diameter per slice for the cut apple slices that ranged from 60 to 75 mm was 6.9 mm (±0.5 mm), and the average mass per slice was 8.8 g (±1.7 g).

The soluble solid of fresh apple ranged from 13.0 to 15.4°Brix measured using a refractometer (model 10430, Reichert-Jung, USA).

#### 2.1.2. Drying

A commercial home dehydrator (model BDA020, 250 W, Blanik, China) with cylindrical shape (25 × 32 cmheight × diameter), five trays, and temperature range from 35 to 70°C [3] was used for drying the sliced fresh “Fuji” apples.

The home dehydrator was operated at 50 and 70°C (nominal temperatures), taking the equipment regulator to such temperatures, with a total sliced apple load of 250 and 500 g, uniformly distributed in five trays. Drying was carried out for 7 hours, and every hour, the trays were rotated, changing their position from the top to the bottom. Only the middle tray was always kept in the same position. The home dehydrator user manual recommends this procedure.

Sliced apple moisture content on a wet basis (w.b.) was monitored every hour during their drying. Fruit from the middle tray was used to measure the moisture content. Therefore, for each experiment, an additional 30 g of sliced apples was placed into the middle tray before beginning the drying time.

### 2.2. Temperature Drying and Electric Energy Consumption

The temperature was monitored with alcohol thermometers (Brannan, UK) placed inside of the three trays at the top, middle, and bottom of the home dehydrator. The temperature measurement by the alcohol thermometer was ensured by contrasting with a calibrated digital thermometer (TM-906A, Lutron Electronic Enterprise Co., Taiwan) and Type K thermocouples, according to the calibration certificate SMD-70491 (8^th^ June 2022) from the Chilean office of Bureau Veritas S.A. (France).

From the beginning to the end of the drying, the electric energy consumption was monitored every hour using a power electric consumption meter model 24050 (as-Schwabe, Germany).

### 2.3. Fruit Analysis

#### 2.3.1. Moisture Content

Between 4 and 5 g of sliced “Fuji” apples was used to determine the moisture content. Fruits were cut in half, put in an oven at 105°C for 2 h, and then weighed. They were kept at 105°C until a constant weight [23].

#### 2.3.2. Fresh Fruit Soluble Solids

Sliced fresh “Fuji” apples were compressed, and the juice obtained was separated, mixed, and measured with a refractometer (model 10430, Reichert-Jung, USA) as °Brix [23].

#### 2.3.3. Ascorbic Acid Content

Sliced fresh (25 g) or dried (10 g) “Fuji” apples were cut in half, subsequently transferred to a 100 mL bottle, and 50 mL of distilled water that had been previously conditioned to a temperature of 30°C was added. The ascorbic acid fruit extraction was performed in an oven (GFL-3032, Germany) under agitation (170 rpm) for 20 min at 30°C [24]. Subsequently, the ascorbic acid reflectometric test strip (Merck, Germany) was immersed for 20 seconds in the aqueous extract, the excess extract was removed, and the concentration determined, introducing the strip for 10 seconds into Merck RQ Flex at 570/657 ± 10 nm [25]. The results were expressed as mg ascorbic acid per 100 g dry weight (d.w.).

#### 2.3.4. Total Polyphenol Content (TPC)

The aqueous extracts from sliced fresh (25 g) or dried (10 g) “Fuji” apples were prepared by the same procedure described in Section 2.3.3. The Folin–Ciocalteu method was used for TPC determination [26]. A 40 *μ*L aliquot of aqueous sliced apple extract was mixed with distilled water (3.16 mL), 200 *μ*L of Folin–Ciocalteu reagent was added, and after 5 min, 600 *μ*L of 20% Na_2_CO_3_ solution was added. Samples were kept at 20°C for 120 min in the dark. The absorbance was measured at 765 nm using a spectrophotometer (Spectronic Genesys 10, Sweden), and the results were expressed as mg of gallic acid equivalents (GAE) per 100 g dry weight (d.w.) using a calibration curve [24].

#### 2.3.5. Color

The color of sliced fresh or dried “Fuji” apples was quantified with a Minolta Chromameter CR-200b colorimeter with the CIE *L*^∗^*a*^∗^*b*^∗^ system as described in Ihl et al. [25]. Ten apple slices were used for each treatment, and the color was measured on both sides of each one. The instrument was calibrated with a white standard tile (*Y* = 93.1, *x* = 0.3140, and *y* = 0.3212) under illuminant condition C (6774 K). The *L*^∗^ variable lightness index ranges in the scale from 0 for black to 100 for white. The *a*^∗^ scale measures the degree of red (+*a*^∗^) or green (−*a*^∗^) colors, and the *b*^∗^ scale measures the degree of yellow (+*b*^∗^) or blue (−*b*^∗^) colors. The dried apple colors were compared with respect to fresh fruit by converting the *L*^∗^*a*^∗^*b*^∗^ values to the difference parameter Δ*E* = (Δ*L*^∗2^ + Δ*a*^∗2^ + Δ*b*^∗2^)^0.5^ [17, 19, 20].

#### 2.3.6. Experimental Design and Statistic Analysis

A completely randomized experimental design was applied to analyze the effects of the drying temperature (50 and 70°C) and mass load (250 and 500 g) of sliced fresh “Fuji” apples in a home dehydrator on moisture content, electric energy consumption, ascorbic acid, total polyphenol content, and color of the dried fruit. The selection of variable levels was based on the conditions reported by Burnham et al. [7], Maheswari [8], Dipersio et al. [9], Weber et al. [10], Kragh et al. [11], Low et al. [12], and the home dehydrator user manual [3]. The experiments were carried out in triplicate, which means that for each treatment (50°C/250 g, 50°C/500 g, 70°C/250 g, and 70°C/500 g), three different samples of sliced dried “Fuji” apples were produced. The data were subjected to analysis of variance, the sources of variance being temperature and mass load. Tukey's honestly significant difference test was used to determine significant differences between treatment means. Mean values were considered significantly different at *p* < 0.05. Minitab® Statistical Software 21.0.3.1.0 (Chicago, USA) was used for data analysis.

## 3. Results and Discussion

### 3.1. Drying Temperature and Mass Load in the Home Dehydrator

**Figure 1:**
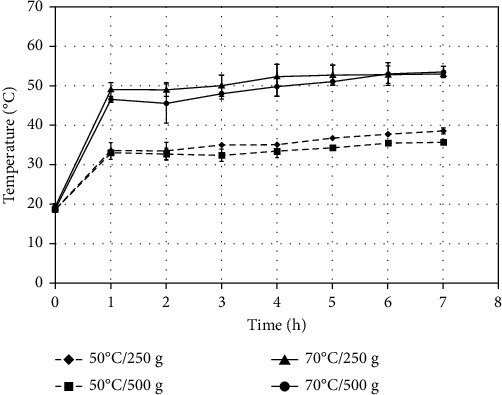
Temperature evolution inside the middle tray of home dehydrator during drying of sliced “Fuji” apples.

Figure 1 shows the temperature evolution inside of the middle tray during the seven hours of home dehydrator operation to dry sliced “Fuji” apples via four treatments. The ambient temperature during experiments at the Food Science Laboratory, Universidad de La Frontera, ranged from 18 to 20°C.

We sought to get as close as possible to the usual procedure for filling a home dehydrator. Therefore, to reach 250 or 500 g, the slices come from normally cutting each apple, and their diameters varied in a range of 60 to 75 mm. However, the larger the diameter of the cut apple slice, the higher is the area and mass per unit. Therefore, for the larger diameter of each apple slice, fewer units of slices will be necessary to complete the mass load of 250 and 500 g of the home dehydrator.

Implicit in Tables 1–3 are the one-way ANOVA with comparison of means by Tukey's honestly significant difference test at *p* < 0.05.

Table 1 presents the average and standard deviation of the maximum temperature for each treatment and the three trays of the home dehydrator evaluated when the sliced apple reached the hour 7 of drying. In Table 1, for each column, which corresponds to one of the four treatments (50°C/250 g, 50°C/500 g, 70°C/250 g, and 70°C/500 g), the effect of the three trays (top, middle, and bottom) is compared statistically among them using lowercase letters, while, for each line, which corresponds to one of the three trays (top, middle, and bottom), the effect of the four treatments (50°C/250 g, 50°C/500 g, 70°C/250 g, and 70°C/500 g) is compared statistically among them using capital letters. For each treatment, significant differences (*p* < 0.05) in the maximum temperature were observed among trays arranged on the top, middle, and bottom of the home dehydrator. As observed in Table 1, during sliced “Fuji” apple drying, the level of nominal temperature (50 and 70°C) was not reached for any of the trays, regardless of the total load of sliced “Fuji” apples (250 or 500 g) placed in the home dehydrator. Probably, the lower ambient temperature (18-20°C) of the laboratory and the lack of thermal insulation of the trays are the causes of not reaching the nominal temperatures. According to Kragh et al. [11], when the sliced mushrooms were dried in a household food dehydrator, they also determined that the air temperature set at 55°C did not reach this value in practice, measuring 46.6°C as the highest dry bulb temperature in the equipment.

There was a nonuniform distribution of the temperature among the positions of the trays of the home dehydrator (Table 1). The equipment has a heating system at its base, so the tray that is positioned at the bottom reached the highest temperature. At the end of drying, the maximum average temperature difference between the bottom and top reached 11°C for 70°C/500 g. This operation condition can certainly affect the drying of the sliced apples. When the dehydrator was operated at 50°C on 250 g of apple, the lowest temperature difference (5°C) was observed between the bottom and top. This is possibly due to less heat losses from the trays to the outside (18-20°C) considering the lower temperature difference between the dehydrator and the surrounding environment. On the other hand, the lower fruit load (250 g at 50°C) would allow a better airflow through the trays. This would favor that the energy generated at the base of the heating system reaches the top tray which improves the temperature distribution among the dehydrator trays. As observed in Figure 1, in general, for the same nominal temperature (50 and 70°C), the load of 250 g showed a real temperature rise higher than the load of 500 g. On the contrary, Weber et al. [10] reported that during drying, there were slightly higher temperatures in the top tray for a higher mass load of chicken pieces in a home-style dehydrator, but the difference between temperatures was not significant (*p* > 0.05).

**Figure 2:**
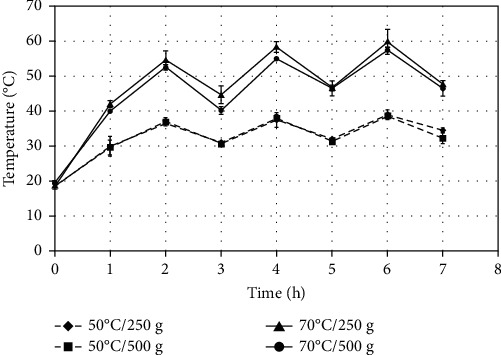
Temperature evolution inside the top tray of home dehydrator at the beginning of drying of sliced “Fuji” apples and their sequential rotation at the bottom position.

**Figure 3:**
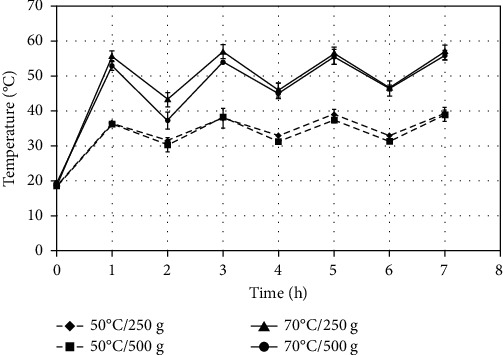
Temperature evolution inside the bottom tray of home dehydrator at the beginning of drying of sliced “Fuji” apples and their sequential rotation at the top position.

As indicated in the methodology (Section 2.1.2), during the 7 hours of drying, the trays were rotated every hour, changing their position from the top to the bottom. Only the middle tray was always kept in the same position; therefore, the fruit in that tray was subjected to the temperature levels that are presented in Figure 1, for each treatment. For the trays that were at the top and bottom at the beginning of drying and proceeded to be rotated, the fruits changed their temperatures sequentially. They increased or decreased the temperature level as the tray was positioned at the bottom or top, respectively, every one hour (Figures 2 and 3).

Table 2 shows the temperature average and standard deviation for those fruits dried in trays of the home dehydrator that were rotated (top and bottom) and kept in the same position (middle). It includes the initial temperature and those during the seven hours of drying for each treatment. In Table 2, for each column, which corresponds to one of the four treatments (50°C/250 g, 50°C/500 g, 70°C/250 g, and 70°C/500 g), the effect of the three trays (top, middle, and bottom) is compared statistically among them using lowercase letters, while, for each line, which correspond to one of the three trays (top, middle, and bottom), the effect of the four treatments (50°C/250 g, 50°C/500 g, 70°C/250 g, and 70°C/500 g) is compared statistically among them using capital letters. For all treatments (Table 2), the sliced “Fuji” apple average temperatures did not differ (*p* > 0.05) among the three trays. However, for each tray, the average temperatures differed (*p* < 0.05) among the four treatments. The highest thermal effect on the fruit was 70°C/250 g.

Temperature affects the chemical and physical characteristics of dried apples, including ascorbic acid, polyphenols, and color [8, 9, 12, 19, 21]. The air-drying temperatures of 40, 60, and 80°C affected the total phenolic content and color of the apple (var. Granny Smith) slices [17]. The effect of temperature and mass load on ascorbic acid, total polyphenol content, and color of the sliced “Fuji” apples evaluated in this work is discussed later.

### 3.2. Fruit Moisture Content Evolution in the Drying

**Figure 4:**
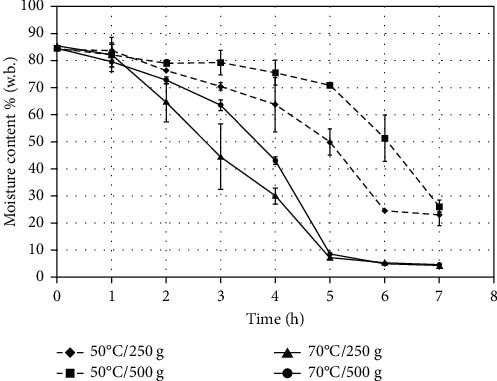
Evolution of moisture content (w.b.) of sliced “Fuji” apples (middle tray) during drying in home dehydrator.

As shown in Figure 4, for monitoring the evolution of moisture content (w.b.) during drying, sliced “Fuji” apples from the middle tray were measured every hour. The initial and end moisture contents (average and standard deviation) of the fruit for each treatment are presented in Table 3.

The effect of the drying temperature (70 and 50°C) and mass load (250 and 500 g) in a home dehydrator showed a different behavior with the decrease of moisture content (84.8%) of sliced fresh “Fuji” apples (Figure 4). After five hours, the fruit dried at the nominal temperature of 70°C, corresponding to the real average temperature (Table 2) of 47.2°C (250 g) and 45.8°C (500 g), presented a moisture content lower than 10%. At the end of drying, 70°C/250 g and 70°C/500 g reached 4.3 and 4.5% (w.b.), respectively (Table 3), while for treatment at the nominal temperature of 50°C, the moisture contents were 23.1 and 26.2% for 250 and 500 g load of the home dehydrator at the end of drying, respectively. Chilean food regulations do not establish moisture level requirements for dried fruits, but Argentina proposes a regulation of the quality and identity of dried fruits at the time of packaging that requires humidity lower than 25% [27]. The Republic of South Africa, via regulation 653, seeks to establish a moisture content limit of <27% for dried fruit intended for sale, which is in relation to the quality, packing, and marketing of products, according to Arendse and Jideani [28]. The four treatments would allow compliance with international requirements of moisture content for dried fruits. According to the Codex Alimentarius [29], the fruit dried as a finished product should be of such moisture content that it can be held in the localities of origin and distribution under any normally foreseeable conditions for those localities without significant deterioration via decay, mould, enzymatic changes, or other causes. The aforementioned international institution is preparing a proposed draft standard for dried fruits, which includes 48 types of fruit, including apples [30].

On the other hand, the shelf life of sliced apple fruit dried by air cabinet dehydrator (Excalibur, model EXC 10) could be improved with moisture content ranging from 6.0 to 7.3% [28]. In another research, apple slices were dried using an oven dryer at 40–50°C until the moisture content left was ≤12%, and after, they were stored at 4°C for 25 days. A positive effect on storage stability was reached when a probiotic (*Bifidobacterium bifidum*) was added to dried apple snacks [31]. Due to low appearance quality and probiotic concentration, Cui et al. [32] reported a nonconvenient storage of apple cubes dried at 40°C for 12 h in a cabinet air drier in which the moisture content was 2.96 ± 0.2% at the end of air drying. In addition, a storage study of the quercetin derivative stability of vacuum-impregnated apple slices after three drying methods considered the fruit with moisture content levels of 6.8% for freeze drying, 9.0% for microwave vacuum drying, and 12.7% for air drying [18]. The air-drying treatment in the home dehydrator at 70°C (Figure 4) allowed the dried apple to reach the moisture contents in the range previously mentioned. In this work, we do not evaluate the shelf life of sliced dried “Fuji” apples obtained at the end of drying of the four treatments because, normally, the home-made fruits are stored in different conditions of packaging, temperature, air humidity, and light incidence. A future study should consider the evaluation and recommendations of dried apple storage made at the home level.

### 3.3. Electric Energy Consumption Behavior

**Figure 5:**
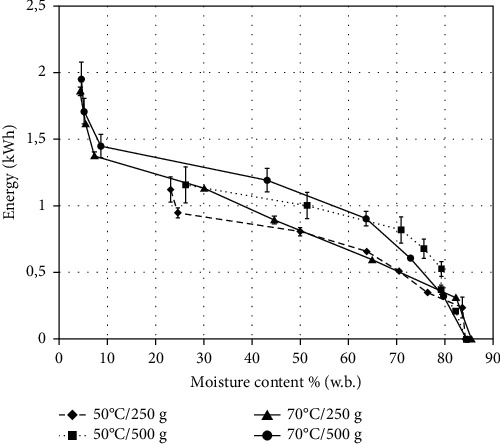
Evolution of electric energy consumption with respect to moisture content (w.b.) of sliced “Fuji” apples (middle tray) during drying in home dehydrator.

After seven hours of drying, the electric energy consumption was 1.123 (±0.094), 1.157 (±0.134), 1.859 (±0.031), and 1.955 (±0.127) kWh for 50°C/250 g, 50°C/500 g, 70°C/250 g, and 70°C/500 g, respectively.  Figure 5 shows the evolution of electric energy consumption during the drying process of the sliced “Fuji” apples for the mentioned treatments. As observed in Table 3, different moisture contents were reached with the electric energy consumed at the end of drying for the four treatments. According to Figure 5, for the temperature of 50°C, a clear difference was observed between the energy consumption curves of the 250 and 500 g dried apples, when their moisture content decreased from 80 to 26% (w.b.). Different behaviors between energy consumption curves of 250 and 500 g for 70°C are also shown in Figure 5, when the fruit moisture content changed from 80 to 10% (w.b.). At both temperatures, the energy consumption of drying 500 g was higher than that of drying 250 g in the mentioned moisture content range.

Filippin et al. [22] established that the application of thermal intermittence in the convective drying of Fuji apple slices (86% (w.b.)) consumed less energy than the continuous drying, with values ranging from 0.812 to 3.385 kWh for intermittent and 3.150 to 4.758 kWh for continuous.

Shewale et al. [33] introduced the concept of the energy consumption rate (ECR) to compare electromagnetic radiation pretreatment (infrared and microwave) before using low humidity air (LHA) and normal hot air (HA) at 40°C for drying apple slices. ECR is the rate between the consumed energy value (kWh) and the amount of moisture removed during drying (kg). The ECR calculated in our experiments were 5.57, 2.88, 8.76, and 4.66 kWh/kg of water removed for 50°C/250 g, 50°C/500 g, 70°C/250 g, and 70°C/500 g, respectively. The energy consumption rates determined for our treatment at 70°C are in agreement with those reported by the recent mentioned authors, which ranged between 3.37 and 5.36 kWh/kg of water removed for LHA drying and 7.09 to 9.65 kWh/kg for HA drying to reduce apple slice (nearly 750 g) moisture content from 82.89-86.15 to 5-6% (w.b.). The treatments 70°C/250 g and 70°C/500 g changed the moisture content from 84.8 to 4.3 and 4.5% (Table 3), respectively, and the average temperature inside of the trays ranged from 44.7 to 47.6°C (Table 2).

A study of a hybrid infrared convective dryer used for drying of apple slices (82% (w.b.)) determined the lowest energy consumption rate of 4.32 MJ/kg (1.2 kWh/kg) for an infrared value of 0.30 W/cm^2^ with air drying at 50°C, while the higher ECR value was 12.72 MJ/kg (3.5 kWh/kg) found at 0.15 W/cm^2^ and 30°C [20]. The lowest ECR (2.88 kWh/kg) calculated for our treatment of 50°C/500 g can be explained by less variation in the moisture content (84.8 to 26.2%) during drying (Figure 5 and Table 3). In this case, more drying time would be required for the dried apple slices to reach the similar moisture content reported by Shewale et al. [33] and Arendse and Jideani [28] ranging from 5 to 7.3% (w.b.).

Probably the home dehydrator used to obtain sliced “Fuji” apples in this research consumes energy levels similar to that produced by an industrial process. However, no comparative studies were made. On the other hand, home drying should preserve apples that are not consumed fresh to, consequently, prevent their waste.

### 3.4. Drying Effect on Fruit

Table 3 presents the effect of the drying temperature and mass load in a home dehydrator on moisture content, ascorbic acid (AA) content, total polyphenol content (TPC), and color of the sliced fresh and dried “Fuji” apples. In Table 3, for each column that corresponds to one of the fresh and dried apples by four treatments (50°C/250 g, 50°C/500 g, 70°C/250 g, and 70°C/500 g), the moisture content, AA, TPC, and color cannot be compared statistically among them, based on the lowercase letters, because they are four different fruit quality parameters. However, for each line which corresponds to one of the four different fruit quality parameters (moisture content, AA, TPC, and color), the effect of fresh and dried apples by four treatments (50°C/250 g, 50°C/500 g, 70°C/250 g, and 70°C/500 g) can be compared statistically among them based on the capital letters.

Changes in moisture content for the four treatments of drying were significant (*p* < 0.05) with respect to the fresh fruit, and their evolution is shown in Figure 4. The main effect was the drying temperature. Although the low mass load (250 g) produced a slightly lower decrease in the moisture content of the fruit compared to 500 g, for the same temperature, it was not significant (*p* > 0.05). The high temperature (70°C) contributed to the heat transfer of air to the fruit for its faster water evaporation [19] to allow a lower moisture content after seven hours of drying with respect to treatment at 50°C.

The ascorbic acid content of freshly sliced “Fuji” apples decreased significantly during drying (*p* < 0.05) as a function of temperature and mass load in the home dehydrator (Table 3). The loss of AA content with respect to fresh fruit was 38.8, 27.7, 40.6, and 54.8% for 50°C/250 g, 50°C/500 g, 70°C/250 g, and 70°C/500 g, respectively. Ertekin and Seydim [21] reported the loss of AA at 70% when fresh Golden Delicious apple slices (16.05 mg AA/100 g d.w.) were dried at 75°C in a rotary forced-air dryer. They evaluated three temperatures (65, 70, and 75°C), and the AA degradation was accelerated with an increase in temperature. The loss of AA was higher at 70 than 50°C in our experiments. Ascorbic acid is thermolabile, highly susceptible to oxidation, and easily degraded. The degradation is accelerated during drying due to high temperature and oxidation [21]. The use of electromagnetic radiation (EMR) pretreatment before drying apple slices with low humidity air retained a higher amount of AA (78.7–67.3%), compared to control and EMR before normal hot air drying (49.4–59.0%), that could be attributed to a shorter drying time and lesser oxidative losses [33]. Those AA retentions are equivalent to AA losses ranging from 21.3 to 50.6%, which are similar to the loss percentages obtained with our drying condition using a home dehydrator. According to Burnham et al. [7], ascorbic acid enhances the destruction of *Escherichia coli* O157:H7 during home-type drying of apple slices after previously immersing in a 3.4% AA solution for 15 min. However, AA concentrations were not informed by those authors. On the other hand, Dipersio et al. [9] established that immersing fruit pieces in 3.4% ascorbic acid solution before home-type dehydration maintained or improved the appearance and overall acceptability of dehydrated apple slices.

The initial total polyphenol content of the freshly sliced “Fuji” apples used in our research (Table 3) is similar to the value of apple var. Granny Smith (158.28 ± 0.65 mg GAE/100 g d.w.) reported by Vega-Gálvez et al. [17]. However, it is lower than the values of Starking Delicious, Golden Delicious, and Granny Smith apples (275-384 mg GAE/100 g d.w.) reported by Ertekin and Seydim [21] and Kala Kullu apple (340 mg GAE/100 g d.w.) reported by Afzaal et al. [31]. Significant decreases (*p* < 0.05) in TPC for the four drying treatments were observed with respect to the fresh fruit. The TPC losses before fruit drying were 26.6, 41.7, 50.6, and 43.7% for 50°C/250 g, 50°C/500 g, 70°C/250 g, and 70°C/500 g, respectively. Similar to AA, the loss of TPC at 70°C was higher than that at 50°C in our experiments. Vega-Gálvez et al. [17] reported that an increase in drying temperature produced higher degradation of total phenolics with respect to content in fresh apples. After drying using a convective dryer at 40, 60, and 80°C, the TPC ranged from 44.82 to 27.04 mg GAE/100 g d.w., which represent a loss of 71.7 to 82.9%, that are higher than the percentages founded in our experiments. Maybe it could be attributed to a difference with respect to apple variety used and drying operation (type of drier, temperature, time, and air velocity). The decrease in polyphenol and anthocyanin contents in fruit after heat treatment is associated with the degradation of the compounds, which generates smaller molecules or polymerized compounds or, in the case of anthocyanins, produces a molecular ring rupture [34, 35], changing the concentration of phenolic compounds when fresh apples are dried by different methods [36].

On the contrary, Ertekin and Seydim [21] reported an increase in TPC in dried apples of the three varieties evaluated; according to the literature mentioned by those authors, the thermal processing could degrade the conjugated phenolic compounds and the release of bound phenolic compounds. In the same sense, during storage of dried apples in the presence of free probiotics, Afzaal et al. [31] informed that after 2 weeks, a rapid increase in the phenolic content was noted, and they attribute the possible degradation of these compounds to the encapsulated bacteria.

Several researches had been evaluating the effect of drying on the color of fresh apples [17–20, 22, 28, 32, 33]. The difference parameter Δ*E* between fresh and dried apples in Table 3 shows that low values were obtained with drying at 70°C, which means that both colors most resemble each other. However, the Δ*E* at 70°C/250 g did not differ significantly (*p* > 0.05) with respect to those Δ*E* values obtained with treatments 50°C/250 g and 50°C/500 g. For drying in a convective dryer at 40, 60, and 80°C, the Δ*E* ranged from 18.75 to 37.17 between fresh and dried apples, and prolonged drying at a higher temperature favored browning reactions that caused a decrease in the lightness (*L*^∗^) value [17]. Similar to our results, the lowest Δ*E* values were obtained with the highest drying temperature. Cruz et al. [19] reported that the fresh apple color was also significantly affected by convective drying (30, 40, 50, and 60°C), resulting in high Δ*E* values between 19.43 and 25.04. However, El-Mesery et al. [20], using a hybrid infrared convective dryer, determined Δ*E* values lower than our results. For air temperatures of 30, 40, and 50°C at an infrared intensity of 0.15, 0.20, and 0.30 W/cm^2^, the Δ*E* ranged from 7.52 to 12.41. Those authors reported that an elevation in Δ*E* values was detected with the rise of air temperature and intensity of radiation.

According to Filippin et al. [22], the parameters *a*^∗^, *b*^∗^, and *C*^∗^ in dried apple samples presented increments in relation to the fresh parameters. These authors consider that long drying time at low temperatures could promote color changes associated with browning product formation in apples. Others results showed that drying with hot air causes a significant color alteration of apple slices [14, 33].

## 4. Conclusion

Home-drying operation (model BDA020, 250 W, Blanik, China) is heterogeneous with respect to temperature behavior among the trays that influence the moisture content of sliced dried “Fuji” apple. However, the procedure of tray position rotation from the top to the bottom and vice versa every hour allows the fruit's average temperature to be uniform.

The treatment at nominal 70°C, regardless of the mass load, makes it possible to reach sliced “Fuji” apple moisture levels below 10% (w.b.) in five hours, which is considered adequate to preserve this fruit as a dried product.

The performance of electrical energy consumption and ECR of the home dehydrator operation used in this work, allowing the moisture content to reach 4.3-4.5% (70°C/250 g and 70°C/500 g) and 23.1-26.2% (50°C/250 g and 50°C/500 g) for sliced “Fuji” apple, is equivalent to that reported for other types of dehydration equipment operated under similar conditions.

The drying conditions at nominal 50°C favored the quality of the sliced dried “Fuji” apples with regard to ascorbic acid content and total polyphenol content; however, the treatment at nominal 70°C produced less variation in color with respect to the fresh fruit.

The use of the home dehydrator evaluated in this work allows obtaining dried slices of “Fuji” apples that adequately preserve the ascorbic acid content, total polyphenol content, and color with respect to the fresh fruit condition.

## Figures and Tables

**Table 1:** Average and standard deviation of maximum temperature (°C) at hour 7 of drying for each treatment and three trays of the home dehydrator.

	50°C/250 g	50°C/500 g	70°C/250 g	70°C/500 g
Top	34.5bB ± 0.7	32.3bB ± 1.5	47.7cA ± 1.2	46.5cA ± 2.1
Middle	38.5aB ± 0.7	35.7abC ± 0.7	53.0bA ± 0.0	53.5bA ± 1.4
Bottom	39.5aB ± 0.7	39.0aB ± 2.0	57.8aA ± 1.8	57.5aA ± 0.7

In each column, different lowercase letters indicate a significant difference by Tukey's honestly significant difference test at *p* < 0.05. In each line, different capital letters indicate a significant difference by Tukey's honestly significant difference test at *p* < 0.05.

**Table 2:** Average and standard deviation temperature (°C) inside of the trays rotated (top and bottom) and kept in the same position (middle), for each treatment of fruit drying.

	50°C/250 g	50°C/500 g	70°C/250 g	70°C/500 g
Top	32.4aB ± 6.5	32.0aB ± 6.4	46.6aA ± 13.0	44.7aAB ± 12.0
Middle	33.6aBC ± 6.4	31.9aC ± 5.5	47.2aA ± 11.7	45.8aAB ± 11.0
Bottom	33.6aB ± 6.8	32.9aB ± 6.7	47.6aA ± 13.0	45.8aAB ± 12.4

In each column, different lowercase letters indicate a significant difference by Tukey's honestly significant difference test at *p* < 0.05. In each line, different capital letters indicate a significant difference by Tukey's honestly significant difference test at *p* < 0.05.

**Table 3:** Characterization of sliced “Fuji” apples after (fresh) and before seven hours of drying using home dehydrator.

	Fresh	50°C/250 g	50°C/500 g	70°C/250 g	70°C/500 g
Moisture content (% (w.b.))	83.5A ± 0.3	23.1B ± 4.1	26.2B ± 2.1	4.3C ± 1.0	4.5C ± 0.1
AA content (mg/100 g d.w.)	32.5A ± 1.3	19.9C ± 0.4	23.5B ± 1.2	19.3C ± 0.0	14.7D ± 1.4
TPC (mg GAE/100 g d.w.)	165.3A ± 6.4	121.4B ± 13.3	96.3C ± 10.7	81.6C ± 2.2	93.1C ± 2.7
Color (Δ*E*)	—	14.0A ± 3.8	13.4A ± 2.7	12.2AB ± 2.8	8.8B ± 1.4

In each line, different capital letters indicate a significant difference by Tukey's honestly significant difference test at *p* < 0.05.

## Data Availability

Data are available on request from the corresponding author.
